# Hommage au Professeur Pierre Pène (1924 - 2023)

**DOI:** 10.48327/mtsi.v4i1.2024.477

**Published:** 2024-01-31

**Authors:** Jean DELMONT

**Affiliations:** Président honoraire de la SFMTSI; Professeur honoraire de la Faculté des Sciences médicales et paramédicales, Aix-Marseille Université

À la suite de son internat et de son clinicat à Bordeaux, le professeur Pène, après avoir été déclaré admissible à l'agrégation de médecine générale, est détaché en octobre 1952 au titre de la coopération universitaire auprès de l’École de médecine de Dakar en tant que professeur suppléant. Sur le plan hospitalier, il est médecin adjoint du professeur Maurice Payet, alors directeur de l’École de médecine. Agrégé de médecine générale en 1958, Pierre Pène est nommé en 1961 professeur titulaire de la chaire de pathologie médicale à la Faculté de médecine et de pharmacie de Dakar, récemment créée. Il travaillera avec le professeur Marc Sankalé, futur doyen, et les professeurs Maxime Armengaud et Michel Rey. Il aura pour élèves les futurs professeurs Auguste Bourgeade et Michel Dumas. Cette époque voit, sous son impulsion, l’émergence de la Société médicale d'Afrique de l'Ouest et de son bulletin. Débute aussi la parution de la revue *Médecine d'Afrique noire* et la tenue périodique de Journées médicales africaines. En 1964, il contribue à la création, au développement et à l'essor de l’École de médecine d'Abidjan dont il est nommé directeur. Il conçoit les plans d'un centre hospitalo-universitaire et deviendra en 1967 le premier doyen de la nouvelle Faculté de médecine.

**Figure 1:**
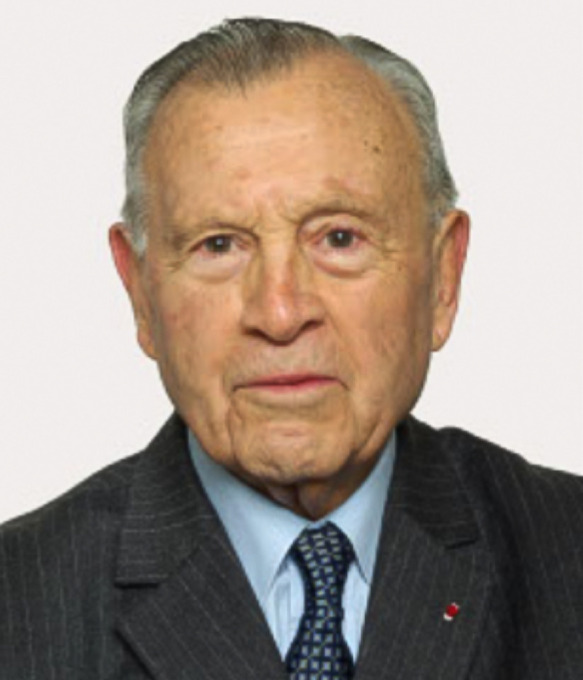
Pierre Pène (archive familiale) Pierre Pène (family archive)

Arrivé à Marseille en 1970, après 19 années de présence en Afrique, il est nommé, l'année suivante, professeur de clinique des maladies tropicales à la Faculté de médecine de Marseille et chef du service de Clinique des maladies exotiques dont l'origine remontait à 1890. L'Institut de médecine et pharmacie tropicales, créé en 1903, venait d’être transformé en une Unité d'enseignement et de recherche (UER) dénommée « Médecine tropicale » dont il fut le directeur. Cette UER bénéficiait d'une autonomie de gestion administrative et financière. Y étaient rattachés les laboratoires de parasitologie, de bactériologie et d'hygiène de la Faculté de médecine. Elle deviendra plus tard le Centre de formation et recherche en médecine et santé tropicales.

Avec l'aide du ministère de la Coopération, Pierre Pène fait rénover l'hôpital de la Calade dans les quartiers nord de Marseille et le transforme en un hôpital de 100 lits. Il sera inauguré le 28 juin 1978 en présence de Madame Simone Veil, ministre de la Santé, et du président Houphouët-Boigny qui financera en partie l'opération et donnera son nom à l'hôpital. Un home d'accueil des médecins africains venus acquérir des spécialités médicales est construit à proximité et portera le nom de « La Maison du Médecin d'Afrique ». De 1980 à 1989 se tiendront chaque année « Les Entretiens de l'hôpital Houphouët-Boigny » abordant des thématiques d'actualité et réunissant les doyens des écoles et facultés de médecine de l'Afrique francophone, occasion d’échanges et de renforcements de liens.

Les relations anciennes, étroites et amicales, de Pierre Pène avec les médecins militaires servant en Afrique tropicale avaient facilité son intégration à Marseille et auguraient d'une collaboration étroite et bénéfique avec l'imposante et prestigieuse École du Pharo qui disposait, entre autres, de centres de recherche et de documentation. Le professeur Pène et ses successeurs ont facilité la reconnaissance universitaire des formations dispensées au Pharo, permettant l'obtention de diplômes; du côté de l'institution civile, les enseignants militaires se sont toujours montrés disponibles pour offrir leur savoir et leur expérience aux étudiants. Le professeur Pène a été invité à siéger au Conseil de perfectionnement de l’École du Pharo et était, de 1973 à 1980, membre du Haut comité consultatif de la médecine militaire.

Expert de l'OMS pour l’éducation médicale et passionné de pédagogie, il introduit à la Faculté de médecine les enseignements par objectifs et les enseignements dirigés des étudiants répartis en petits groupes. Il coordonne les enseignements de plusieurs diplômes d'université parmi lesquels le « Diplôme de Médecine et santé tropicales ».

Sollicité pour accompagner l’émergence et le développement d’écoles et de facultés de médecine dans les pays africains, le professeur Pène conclut des accords de coopération avec les universités de Dakar, Abidjan, Bamako, Niamey, Bangui, Yaoundé, Libreville et Tananarive, les pourvoyant d'enseignants français et promouvant la carrière universitaire des médecins locaux les plus méritants. Au cours de l'un de ses nombreux déplacements en Afrique subsaharienne, alors que l'avion d'UTA reliant Bangui à Paris, en retard sur l'horaire, stationnait sur l'aire de l'aéroport de N'Djamena avec ses passagers restés en cabine, explose une bombe placée dans la soute. Pierre Pène en gardera des séquelles respiratoires par émanation de gaz toxiques mais échappera à une mort inéluctable, car l'explosion avait été programmée pour se produire en plein vol au-dessus du désert.

Pour mieux valoriser la médecine tropicale auprès des pouvoirs publics et lui faire reconnaître la place qu'elle mérite, il a fondé avec les instituts parisiens des professeurs Marc Gentilini et Jean-Pierre Coulaud ainsi que l'Institut de médecine tropicale du Service de santé des armées, le Conseil des Instituts français de médecine tropicale, plus tard complété par l'Institut René Labusquière de Bordeaux, dirigé par le professeur Michel Le Bras. En concertation avec d'autres pays européens, il crée un Conseil des Instituts européens de médecine tropicale. Il a aussi contribué à réunir, au sein d'une même section du Comité consultatif des universités, les entités de maladies infectieuses et de maladies tropicales, à l'origine de l'Association des professeurs de pathologies infectieuses et tropicales (APPIT) qui deviendra plus tard le Collège des universitaires des maladies infectieuses et tropicales (CMIT).

Le professeur Pène a orienté les activités de son adjoint, le professeur Auguste Bourgeade, disparu le 5 avril 2022, vers les pathologies et la santé des expatriés, des immigrés et des voyageurs. Celui-ci créera l'un des premiers logiciels de conseils de prévention, EDISAN, et fondera aussi avec les professeurs Armengaud et Rey, l'Association pour la prévention des maladies des voyageurs (ARMAVOY) qui deviendra la Société de médecine du voyage (SMV).

Retraité de l'Université au 1^er^ octobre 1993, Pierre Pène n'a plus voyagé en Afrique pour des raisons de santé, réservant ses déplacements pour participer, à Paris, aux activités de l'Académie nationale de médecine dont il était membre. Désireux de continuer à se rendre utile pendant sa retraite, il avait devancé la date du début de celle-ci par son élection en 1989 comme maire de la ville de Carry-le-Rouet, mandat exercé jusqu'en 2013. Pour quelques années, il fut également vice-président de la Communauté urbaine Marseille Provence Métropole. Malgré ses charges administratives, il continuait à montrer un grand intérêt pour la santé publique des collectivités en France et le développement des pays africains.

Le professeur Pène a profondément marqué de son empreinte l'aide médicale française à la santé des populations des pays d'Afrique francophone, dans la période précédant les indépendances de 1960 et celle des 30 premières années de coopération médicale. Il a formé des milliers de médecins et autres professionnels de santé, africains et français, devenus enseignants pour certains. Il a aussi orienté et accompagné le déroulement de la carrière professionnelle de nombreux élèves. Avec l'aide de ses collaborateurs, il a dirigé ou présidé plus de 300 thèses de médecine, participé à la conception ou l’écriture de près de 500 articles scientifiques, rédigé en collaboration plusieurs ouvrages, et créé 5 revues médicales.

Travailleur infatigable, clairvoyant, agissant avec diplomatie, ses talents pour concevoir, bâtir, créer et organiser ont été reconnus et récompensés par l'attribution des plus hautes distinctions honorifiques, tant dans les pays africains qu'en France où il a été gratifié des titres de commandeur de la Légion d'Honneur, commandeur de l'Ordre national du Mérite, et commandeur de l'Ordre des Palmes académiques.

Homme énergique, volontaire, déterminé, engagé, exigeant avec lui-même et ses collaborateurs, il savait aussi être affable, empathique, respectueux, courtois et bienveillant avec tous ceux qui ont eu l'opportunité et le plaisir de l'approcher.

Le 12 juillet 2023 ont eu lieu à Carry-le-Rouet ses obsèques. Avant la cérémonie religieuse et son inhumation, les honneurs lui ont été rendus sur la place publique pour son passé de résistant pendant la Seconde Guerre mondiale, et son mandat de maire exercé dans cette ville. Ont été prononcés des éloges relatifs à sa carrière hospitalo-universitaire, provenant d'institutions dont il fut membre (Académie nationale de médecine, Académie des sciences d'outre-mer) ou de sociétés savantes, telle la Société francophone de médecine tropicale et santé internationale, ex-Société de pathologie exotique dont, de janvier 1991 à décembre 1994, il assura la présidence. Étaient également représentées plusieurs associations : Amicale Santé Navale et d'Outre-Mer (ASNOM), les Anciens et Amis du Pharo, et les Amis du Patrimoine médical de Marseille.

Son épouse, ses enfants, ses petits-enfants et ses 16 arrière-petits-enfants peuvent être fiers de son exceptionnelle carrière professionnelle et de son œuvre.
